# Variability of Scots pine (Pinus sylvestris L.) plus trees in the Middle and Upper Volga Region with the use of ISSR markers

**DOI:** 10.18699/vjgb-24-17

**Published:** 2024-04

**Authors:** O.V. Sheikina, E.M. Romanov

**Affiliations:** Volga State University of Technology, Yoshkar-Ola, Russia; Volga State University of Technology, Yoshkar-Ola, Russia

**Keywords:** Pinus sylvestris L., plus trees, genetic diversity, differentiation, ISSR markers, Pinus sylvestris L., плюсовые деревья, генетическое разнообразие, дифференциация, ISSR-маркеры

## Abstract

One of the serious issues in forest breeding is how to reduce the variability level in breeding populations of forest tree species that is a set of selected plus trees. The problem is that variability is jeopardized by the risk of losing the genetic diversity of future artificial forests, as well as emerging inbreeding depression in the seed plus trees progeny. DNA markers are an effective tool to study variability, identify features of the genetic structure and degree of plant differentiation. The research focuses on assessing the level of the genetic diversity and the degree of differentiation of plus trees of various geographic origin with the use of ISSR markers. We used six ISSR primers to study 270 plus trees grown in the Penza region, the Chuvash Republic, the Republic of Tatarstan and the Mari El Republic. The samples of plus trees under study were characterized by different levels of genetic diversity. Two hundred fifteen PCR fragments were identified for six ISSR primers in total, while the number of amplified fragments varied from 186 to 201 in different plus trees samples. The genetic variability varied within the following limits: 95.7–96.9 %, polymorphic loci; 1.96–1.97, the number of alleles per locus; 1.31–1.48, the number of effective alleles per locus: finally, 0.291–0.429, Shannon’s index; 0.205–0.298, the expected heterozygosity. According to the analysis of molecular variance (AMOVA), 82 % of the variability of ISSR markers is typical for the plus tree samples, while only 18 % is variability among the compared groups of trees from different geographical zones. The dendrogram generated by UPGMA showed that the plus trees grown in the Penza region, the Chuvash Republic and the Republic of Tatarstan are similar in term of the genetic structure of plus trees, while the plus gene pool of Scots pine from the Mari El Republic stands alone. The results of the research prove that the level of genetic diversity, the structure of genetic variability, and the nature of differentiation of plus trees are consistent with those previously elicited for natural populations of Scots pine in the Middle and Upper Volga region.

## Abstract (ru)

Снижение уровня изменчивости селекционных популяций лесных древесных видов, представляющих собой совокупность отобранных плюсовых деревьев, считается одной из ключевых проблем в лесной селекции. Она связана с опасностью потери генетического разнообразия будущих искусственно созданных лесов, а также с риском возникновения инбредной депрессии семенного потомства плюсовых деревьев. Эффективным инструментом для изучения изменчивости, определения особенностей генетической структуры и степени дифференциации растений являются ДНК-маркеры. Наше исследование направлено на оценку уровня генетического разнообразия и степени дифференциации плюсовых деревьев разного географического происхождения с применением ISSR-маркеров. С использованием шести ISSR-праймеров изучено 270 плюсовых деревьев из Пензенской области, Чувашской Республики, Республик Татарстан и Марий Эл. Сравниваемые выборки характеризовались разным уровнем генетического разнообразия. Всего для шести ISSR-праймеров обнаружено 215 ПЦР-фрагментов, при этом у разных выборок число амплифицированных фрагментов варьировало от 186 до 201. Основные показатели генетической изменчивости находились в следующих пределах: доля полиморфных локусов 95.7–96.9 %, число аллелей на локус 1.96–1.97, число эффективных аллелей 1.31–1.48, индекс Шеннона 0.291–0.429, ожидаемая гетерозиготность 0.205–0.298. По результатам анализа молекулярной дисперсии (AMOVA) установлено, что 82 % вариабельности ISSR-локусов обнаруживается внутри выборок плюсовых деревьев и только 18 % приходится на изменчивость между сравниваемыми группами деревьев из разных географических районов. Построение UPGMA-дендрограммы пока- зало близость генетической структуры плюсовых деревьев из Пензенской области, Чувашской Республики и Республики Татарстан и обособленность плюсового генофонда сосны обыкновенной из Республики Марий Эл. Результаты исследований указывают на то, что уровень генетического разнообразия, структура генетической изменчивости и характер дифференциации плюсовых деревьев соответствуют ранее выявленным для природных популяций сосны обыкновенной в Среднем и Верхнем Поволжье.

## Introduction

Mass selection of plus trees constitutes the Russian selective seed production of the main forest-forming species (Tarakanov et al., 2021). A number of phenotypic characteristics, such as height, diameter, trunk quality, disease resistance, etc., are critical for plus trees selection in natural plantations. First-order tree gene banks, an integral part of the forest-seed establishment, serve for the mass production of seeds of forest tree species by the vegetative offspring of plus trees (Tsarev et al., 2021). One of the concerns, while introducing forest seed programs for Scots pine based on the principles of plus selection, is that the genetic diversity of the plus gene pool declines. This happens due to the selection of a limited number of plus trees, as well as the risk of inbreeding depression of seed offspring that are in proximity to related clones in tree gene banks (Koelewijn et al., 1999; Hosius et al., 2006). Therefore, further studies are necessary to research the diversity of the selected plus gene pool and identify the nature of its differentiation using both morphometric characters (Tarakanov, Kalchenko, 2015; Besschetnova, Besschetnov, 2017) and molecular markers (Shigapov, 1995; Milyutina et al., 2013; Ilinov, Raevsky, 2021).

Molecular markers have become an effective tool that solves a wide range of issues in the field of forest selection and seed production, as well as estimates the genetic diversity of plus trees (Sheikina, 2022b). To assess the variability of Scots pine plus trees and tree gene banks established by their offspring, different researchers used isoenzymes (Shigapov, 1995), ISSR markers (Milyutina et al., 2013; Khanova et al., 2020) and microsatellites (Ilinov, Raevsky, 2021; Kamalov et al., 2022). The results of comparative studies of the genetic diversity of the plus gene pool of tree species and natural populations showed contradictory results. A number of works note that plus trees can be characterized by a level of genetic variability comparable to natural populations (Bergman, Ruetz, 1991; Ilyinov, Raevsky, 2023). On the other hand, we may witness a decrease in allelic diversity in samples of plus trees growing in tree gene banks (Shigapov, 1995; Ilyinov, Raevsky, 2017).

Until now, in the Middle and Upper Volga regions, there have been studies of ISSR loci polymorphism only for a small sampling of 36 plus trees in the Republic of Mari El (Milyutina et al., 2013). However, no assessment of the genetic diversity of Scots pine plus trees in other parts of the Middle and Upper Volga region has been made. Meanwhile, ISSR markers are widely employed to study the characteristics of the population genetic structure of Scots pine in China (Hui-yu et al., 2005), Portugal (Cipriano et al., 2013), on the East European Plain and in the Urals (Vidyakin et al., 2015; Vasilyeva et al., 2021; Chertov et al., 2022; Sboeva et al., 2022), in the Perm Territory (Prishnivskaya et al., 2019) and in the Volga region (Sheikina, 2022a).

The objective of this paper is to study the genetic variability and differentiation of Scots pine plus trees from the Middle Volga region based on the analysis of ISSR markers. We assumed that the level of genetic diversity, the structure of genetic variability and the nature of differentiation of plus trees selected as a result of breeding is comparable to those previously identified for natural populations of Scots pine in the Middle and Upper Volga region.

## Materials and methods

The object of the study was plus trees of Scots pine (Pinus sylvestris L.) or their clones from four regions of the Middle and Upper Volga region. Samples for molecular genetic research in the Republic of Tatarstan were stored from plus trees growing on the territory of the Zelenodolsk forestry. The remaining samples were stored from clones of plus trees growing at forest seed production facilities: in the Chuvash Republic from a first-order tree gene bank in the Ibresinsky forestry, in the Penza Region from a first-order tree gene bank in the Chaadayevsky forestry, in the Mari El Republic from a collection and uterine plot in the Sernursky forestry. In total, the authors studied 270 trees.

The material for DNA extraction was dried pine needles. The CTAB technique (Doyle J.J., Doyle J.L., 1987) was employed for DNA preparations. Six ISSR primers were used for PCR: (СA)6AGCT, (СA)6AG, (CA)6GT, (CA)6АC, (AG)8T and (AG)8GCT (Hui-yu et al., 2005). PCR was carried out in a MJ MiniTM Gradient Thermal Cycler (Bio-Rad, USA) according to the following program: 94 °C – 5 min; 35 cycles: 94 °С – 45 s, 60 °С – 45 s, 72 °С – 45 s; 72 °C – 7 min. To perform PCR, we used the components of the commercial set Encyclo Plus PCR kit (Evrogen, Russia) with the following concentration: 10 × PCR buffer – 1 μl; dNTPs – 0.2 μl (10 mM); primer – 0.1 μl (100 μM); DNA preparation – 1 μl (20 ng); Taq polymerase – 0.1 μl (2 units/μl); water – 7.6 μl. PCR for each sample was performed in triplicate to check the repeatability of the DNA fingerprints obtained. PCR results were visualized with the use of electrophoresis in a 1.5 % agarose gel in 1 × TBE buffer at an electric field voltage of 80 V and staining with an ethidium bromide solution. Gel images were obtained using the GelDoc 2000 gel documentation system (Bio-Rad, USA) and the Quantity One® Version 4.6.3 software package. The ‘100 bp+3.0 kb DNA Ladder’ marker (Evrogen, Russia) was used to calculate the lengths of PCR fragments.

Interpretation of the results of molecular genetic analysis was based on compiling a binary matrix, in which PCR fragments present in the electropherogram were designated as ‘1’, and those absent, as ‘0’. Calculation of genetic diversity indicators, analysis of molecular variance (AMOVA) and principal coordinate analysis (PCoA) were done in the GenAlEx program (Peakall, Smouse, 2012). The statistical significance of differences between the average values of genetic diversity indicators of samples of plus trees was assessed using single-factor analysis of variance. A dendrogram illustrating the genetic relationship of samples of plus trees was drawn based on the frequency of occurrence of ISSR loci in the POPTREEW program (Takezaki et al., 2014) using the Unweighted Pair-Group Method with Arithmetic Mean (UPGMA) with bootstrap support for 10,000 replications

## Results

The authors identified 215 amplified DNA fragments for six ISSR primers, 99.5 % of which turned out to be polymorphic (Table 1). For samples of plus trees of different geographical origins, the number of PCR fragments varied from 186 in the Republic of Mari El to 201 in the Penza Region, and the percentage of polymorphic loci ranged from 95.7 to 96.9. The number of rare PCR fragments with an occurrence frequency of less than 5 % in different samples varied from 1 to 23, and the number of unique ones, from 0 to 2.

**Table 1.**
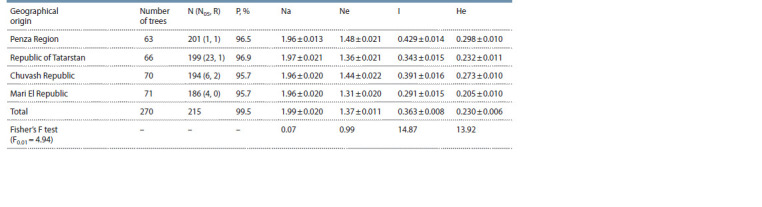
Indicators of genetic diversity of Scots pine plus trees Note. Mean ± standard error. N – number of PCR fragments; N05 – number of PCR fragments with a frequency <5 %; R – number of unique PCR fragments; P – percentage of polymorphic loci; Na – number of alleles per locus; Ne – number of effective alleles; I – Shannon index; He – expected heterozygosity.

**Fig. 1.**
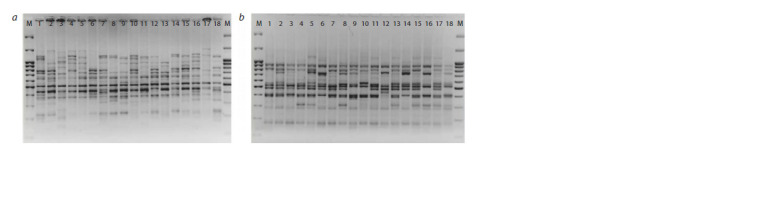
DNA profiles showing polymorphism of Scots pine plus trees obtained with ISSR primers (СA)6AGCT (a) and (AG)8T (b). 1–18 – DNA sample numbers, M – DNA length marker 100 bp + 3.0 kb DNA Ladder.

Shannon information index and expected heterozygosity were different for the studied sample of plus trees. Plus trees from the Mari El Republic proved to have the lowest values of indicators (I = 0.291, He = 0.205). While pine from the Penza Region showed the maximum values of genetic variability (I = 0.429, He = 0.298). The differences between the samples are significant (p = 0.01). In terms of the number of alleles per locus and the number of effective alleles, plus trees of different geographical origins did not differ (Na = 1.96–1.97, Ne = 1.31–1.48) at р = 0.01. In total, for all the trees studied, the number of alleles per locus was 1.99, the number of effective alleles was 1.37, the Shannon index was 0.363, and the expected heterozygosity was 0.230. Figure 1 exemplifies the spectra of PCR fragments.

The different ISSR primers used in PCR allowed us to analyze from 27 to 40 loci, 80.6–93.5 % of which are polymorphic (Table 2). The high level of polymorphism suggests that the studied set of markers can be a useful and informative tool in assessing the genetic variability of both natural populations of an economically valuable species, as well as forest crops and objects of a genetic breeding complex, including plus trees. Other indicators of genetic diversity for different ISSR primers varied in the following ranges: the number of alleles per locus, from 1.62 to 1.90; the number of effective alleles, from 1.31 to 1.41; the Shannon index, from 0.331 to 0.393; the expected heterozygosity, from 0.206 to 0.252.

**Table 2.**
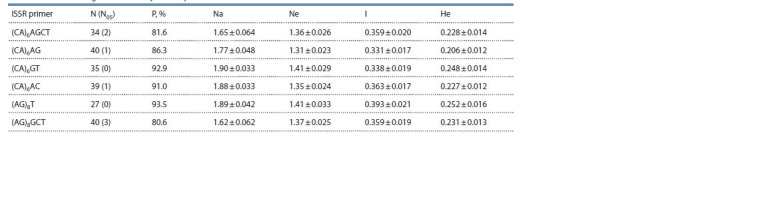
Indicators of genetic diversity of ISSR primers Note. Mean ± standard error. N – number of PCR fragments; N05 – number of PCR fragments with a frequency <5 %; P – percentage of polymorphic loci; Na – number of alleles per locus; Ne – number of effective alleles; I – Shannon index; He – expected heterozygosity.

Analysis of molecular variance proved that 82 % of genetic variability is distributed within samples of plus trees from different geographical areas of the Middle Volga region (Table 3). Interbreeding population variation accounts for 18 % of genetic diversity. Pairwise comparisons of plus trees from different geographic areas showed that interbreeding population variation could account for 14 to 24 %. The greatest genetic subdivision is characterized by samples from the Penza Region and the Mari El Republic (24 %), as well as from the Chuvash Republic and the Mari El Republic (23 %). The share of interpopulation variability was 14–16 % in the remaining cases. In all cases, the significance level was р < 0.001.

**Table 3.**
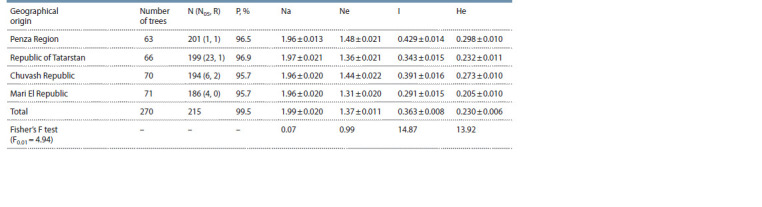
Distribution of intra- and interbreeding population genetic variability of Scots pine plus trees according to the analysis results of molecular variance Note. df – number of degrees of freedom; SS – sum of squares; MS – standard deviation; V – dispersion.

**Fig. 2.**
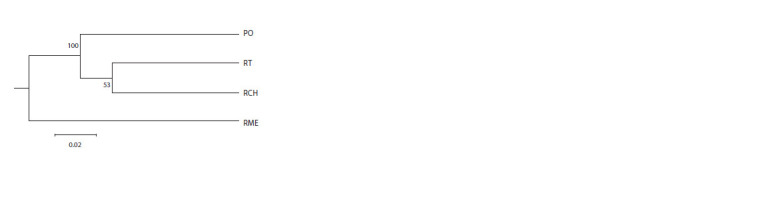
UPGMA dendrogram drawn with NEI’s genetic distance between plus trees of P. sylvestris L. PO – Penza Region, RT – Republic of Tatarstan, RCH – Chuvash Republic, RME – Mari El Republic

Samples of trees from the Penza Region, Republic of Tatarstan and Chuvash Republic were included in one cluster on the UPGMA dendrogram with a high bootstrap value (100) (Fig. 2). The sample of plus trees from the Mari El Republic was assigned to a separate cluster.

**Fig. 3.**
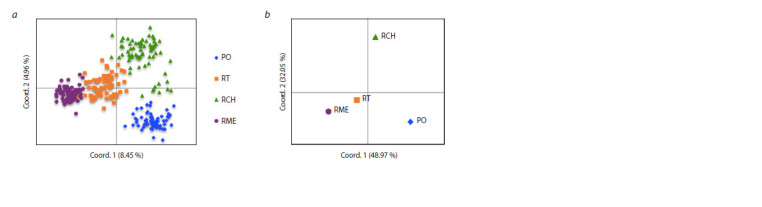
Spatial location of the principal coordinate (PCoA) of Scots pine plus trees (a) and geographic origins (b). PO – Penza Region, RT – Republic of Tatarstan, RCH – Chuvash Republic, RME – Mari El Republic.

The authors analyzed the principal coordinates for individual Scots pine trees (Fig. 3, a) and samples of plus trees (Fig. 3, b) based on pairwise Nei’s genetic distances. Analysis of the principal coordinates for individual Scots pine trees showed that the three principal axes account for 17.03 % of the polymorphism of ISSR loci, with the first coordinate accounting for 8.45 % and the second for 4.96 % of the total variability. At the same time, 81.02 % of the total diversity occurs in the first and second coordinates at the level of plus tree samples. The authors did not identify any geographic gradients along the axes. However, one can note the similarity in the distribution of samples on the first axis with the location of the sampling areas of plus trees in relation to the river Volga along the first axis. Plus trees from the Mari El Republic and Republic of Tatarstan grow on the left bank, while those from the Penza Region and Chuvash Republic grow on the right one

## Discussion

The paper discusses the genetic variability and differentiation of the plus gene pool of Scots pine from different regions of the Middle and Upper Volga region. To preserve the genetic diversity of a species in the process of artificial regeneration, it seems to be crucial that breeding populations are highly variable. Literature review showed that the percentage of polymorphic ISSR loci in Scots pine populations may vary from 42 to 100 % (Hui-yu et al., 2005; Cipriano et al., 2013; Vidyakin et al., 2015; Prishnivskaya et al., 2019). The percentage varied between 95.7–96.9 % and averaged 99.5 % for the studied samplings of plus trees and the selected markers, which aligns with the results of the previous studies. The value the authors got for the proportion of polymorphic loci of plus trees was comparable to the data acquired for 12 natural populations of Scots pine (96.7 %) from the Upper and Middle Volga region, studied with the same set of ISSR markers (Sheikina, 2022a)

Other indicators of genetic diversity identified among the studied samples of plus trees were not inferior to the values typical for natural populations. Thus, the values of the number of effective alleles and expected heterozygosity for plus trees were 1.31–1.48 and 0.205–0.298, respectively, while for natural populations these were 1.27–1.39 and 0.174–0.241 (Sheikina, 2022a). A similar value of the expected heterozygosity of ISSR loci (He = 0.239) was identified for plus Scots pine trees from the Republic of Bashkortostan (Khanova et al., 2020). Lower values of expected heterozygosity were determined for Scots pine populations on the Russian Plain (He = 0.046–0.239) (Vidyakin et al., 2015; Prishnivskaya et al., 2019; Vasilyeva et al., 2021; Sboeva et al., 2022) and in the Urals (He = 0.149–0.185) (Chertov et al., 2022). High values of expected heterozygosity (He = 0.447–0.488) were typical for Portuguese populations (Cipriano et al., 2013), 1.5–2.4 times higher than the values described above.

For the samplings of plus trees under study, the Shannon index varied from 0.331 to 0.393. In other studies, the Shannon index identified for populations from different parts of Russia was 0.087–0.357 (Vasilyeva et al., 2021; Chertov et al., 2022; Sboeva et al., 2022). Higher values of the Shannon index (I = 0.636–0.681) were determined for Scots pine populations from Portugal (Cipriano et al., 2013). Differences in levels of genetic diversity may be explained by both geographic variability and the fact that studies have used different sets and numbers of ISSR markers.

Based on the analysis of the molecular dispersion of plus pine trees of different geographical origins, the authors found that that 18 % of the variability of ISSR loci accounts for the interpopulation component. These data comply with the information previously acquired for natural populations of Scots pine in the Upper and Middle Volga region (14 %) (Sheikina, 2022a). The value of this parameter was 37–48 % (Vasilyeva et al., 2021; Chertov et al., 2022; Sboeva et al., 2022) for the populations from the East European Plain and the Urals. Assessment of the differentiation of Scots pine populations from various parts by measuring the indicator of genetic subdivision (Gst) proved that the interpopulation component of the variability of ISSR loci can account for from 5.8 to 55.8 % (Hui-yu et al., 2005; Cipriano et al., 2013; Vidyakin et al., 2015; Vasilyeva et al., 2021; Chertov et al., 2022; Sboeva et al., 2022; Sheikina, 2022a). Relatively low values of the genetic subdivision indicator were found for populations from the Middle Urals (Gst = 0.155) (Sboeva et al., 2022) and from Portugal (Gst = 0.058) (Cipriano et al., 2013). Higher values of the genetic subdivision indicator were shown for populations from China (Gst = 0.396) (Hui- yu et al., 2005), from the East European Plain (Gst = 0.439– 0.558) (Vidyakin et al., 2015; Vasilyeva et al., 2021; Sboeva et al., 2022) and from the Urals (Gst = 0.362) (Chertov et al., 2022). The indicator of genetic subdivision of natural populations from the Upper and Middle Volga region was 0.161 (Sheikina, 2022a). Thus, the data on the structure of genetic variability in samples of plus trees acquired in this study do not contradict previously described results for natural populations of Scots pine.

Clustering of plus trees samples with the UPGMA method showed the isolation of the plus gene pool of Scots pine from the Mari El Republic from three other groups of trees. Tree samples from the Penza Region, Chuvash Republic and Republic of Tatarstan constitute a single cluster with a similar genetic structure. While assessing the population structure of pine forests in the Middle and Upper Volga regions, the authors also traced the differences between populations growing in the Mari El Republic, on the right Volga riverbank, from left-bank populations growing in the Chuvash Republic and the Penza Region (Sheikina, 2022a). The identified differentiation of populations and plus gene pools of Scots pine of different geographical origins may be the result of the intersection of the species’ migration routes in the post-glacial period. Specifically, with the allozyme analysis, the authors discovered that five different Pleistocene refugia could have participated in creating the gene pool of Scots pine populations on the East European Plain (Sannikov et al., 2020).

## Conclusion

The studied plus trees samples taken from various parts of the Middle and Upper Volga regions differ in the level of polymorphism of ISSR loci. A level of genetic diversity of the plus gene pool of Scots pine selected during breeding is comparable to natural populations in the region under the study. The structure of genetic variability and the nature of differentiation of samples of plus trees of various geographical origin also correspond to the population genetic structure of natural populations.

The authors’ results proved that the ISSR markers described by A.I. Vidyakin et al. (Vidyakin et al., 2015) are feasible for the study of the population genetic structure of Scots pine. Moreover, the high level of variability of the selected loci (80.6–93.5 %) allows recommending this set for assessing the genetic variability of natural populations, forest crops and objects of the unified genetic breeding pool (UGBP). Further studies with the use of other types of molecular markers are necessary to improve the reliability of genetic diversity assessment and differentiation of plus trees.

## Conflict of interest

The authors declare no conflict of interest.
